# Rapid breeding of parthenocarpic tomato plants using CRISPR/Cas9

**DOI:** 10.1038/s41598-017-00501-4

**Published:** 2017-03-30

**Authors:** Risa Ueta, Chihiro Abe, Takahito Watanabe, Shigeo S. Sugano, Ryosuke Ishihara, Hiroshi Ezura, Yuriko Osakabe, Keishi Osakabe

**Affiliations:** 1 Graduate School of Advanced Technology and Science, Tokushima University, Tokushima, Japan; 2 Center for Collaboration among Agriculture, Industry, and Commerce, Tokushima University, Tokushima, Japan; 3 Graduate School of Life and Environmental Sciences, University of Tsukuba, Tsukuba, Japan; 4 Faculty of Bioscience and Bioindustry, Tokushima University, Tokushima, Japan

**Keywords:** Genetic engineering, Molecular engineering in plants, Plant breeding

## Abstract

Parthenocarpy in horticultural crop plants is an important trait with agricultural value for various industrial purposes as well as direct eating quality. Here, we demonstrate a breeding strategy to generate parthenocarpic tomato plants using the CRISPR/Cas9 system. We optimized the CRISPR/Cas9 system to introduce somatic mutations effectively into *SlIAA9*—a key gene controlling parthenocarpy—with mutation rates of up to 100% in the T0 generation. Furthermore, analysis of off-target mutations using deep sequencing indicated that our customized gRNAs induced no additional mutations in the host genome. Regenerated mutants exhibited morphological changes in leaf shape and seedless fruit—a characteristic of parthenocarpic tomato. And the segregated next generation (T1) also showed a severe phenotype associated with the homozygous mutated genome. The system developed here could be applied to produce parthenocarpic tomato in a wide variety of cultivars, as well as other major horticultural crops, using this precise and rapid breeding technique.

## Introduction

In plants, fruit plays the important role of protecting embryos and seeds during development; the vital processes of fruit development, including fertilization, are temporally and spatially controlled by phytohormones^1,2^. Parthenocarpy—the production of seedless fruit without prior fertilization^3–5^—is an important trait in horticultural crop plants, conferring various agricultural benefits. Since fruit growth, including pollination and fertilization, is strongly affected by adverse environmental conditions^3,4,6^, parthenocarpy is advantageous for stable crop production in fluctuating environments. Seedless fruits also have great added value for industrial purposes, e.g., in sauce production.

Plant hormones regulate plant growth and development at all stages, and are controlled by internal signal transduction and environmental cues. The fertilization-independent fruiting and seed development needed for parthenocarpy are controlled by the cross-talk of phytohormones^2,7^. Parthenocarpy is initiated by auxin, and the underlying molecular mechanisms have been extensively characterized^8–12^. The protein factors AUXIN-INDUCED (Aux/IAA) and AUXIN RESPONSE FACTORS (ARFs) are the key regulators in auxin signaling, in which the auxin receptor TIR1 enhances degradation of the negative regulator Aux/IAAs via the 26S proteasome pathway to activate the auxin transcription factor ARF by inhibiting its interaction with Aux/IAA^13,14^. Among the large number of AUX/IAAs and ARFs, Aux/IAA9 (IAA9) and ARF8 are involved in tomato fruit development to repress fruit initiation without fertilization^8,9^. Genetic parthenocarpy identified by classical breeding has been used in only a limited numbers of species and cultivars. More recently, the emergence of genome editing technologies enabling direct and precise genome modification have allowed gene replacement or target-specific gene insertion via homologous recombination, and site-specific gene deletion or insertion via non-homologous end joining on the target of interest by using custom engineered nucleases^15^. The most widespread genome editing technology in current use is the clustered regularly interspaced short palindromic repeat (CRISPR)/CRISPR-associated protein-9 nuclease (Cas9) system, which consists of Cas9 nuclease and a guide RNA (gRNA) that targets the genomic sequence of interest^16,17^. The efficacy of the CRISPR/Cas9 system has been proven in both model plants and many crop plants, such as rice, sorghum, wheat, maize, sweet orange, soybean, potato, apple, and tomato^18–20^. The CRISPR/Cas9 system efficiently introduces site-directed mutations in regenerated plants (T0) as well as later generations^21–25^; however, CRISPR/Cas9 sometimes causes mosaic mutations and off-target effects in various plants. To establish new breeding techniques, with reduced costs and breeding time compared to classical techniques, it is important to develop and optimize highly efficient editing systems for important crop plants.

Here, we applied the CRISPR/Cas9 system to the production of parthenocarpic tomato plants via disruption of the tomato (*Solanum lycopersicum*) *IAA9* (*SlIAA9*) gene in both Micro-Tom and the commercial cultivar Ailsa Craig. Introduction of the mutation was highly efficient, with almost 100% of genomic DNA isolated from tomato cells of the regenerated generation (T0) having the desired mutation, with no off-target mutations. Tomato plants with the *SlIAA9* gene knocked out by our CRISPR/Cas9 system exhibited simple leaves instead of wild-type compound leaves, and fruit development was triggered before fertilization, giving rise to parthenocarpy. The mutations were heritable in subsequent generations. These results suggest that precise and effective genome editing can be used to guide molecular breeding in tomato.

## Results

### CRISPR/Cas9 constructs and design of target gRNA

**Figure 1:**
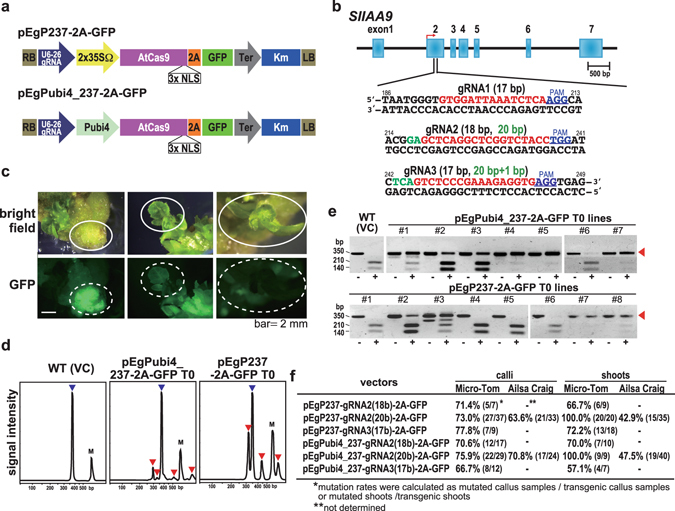
Site-directed mutagenesis in *SlIAA9* using CRISPR/Cas9. (**a**) Schematic representation of T-DNA regions of the CRISPR/Cas9 binary vectors used in this study. U6-26 gRNA: Arabidopsis *U6 snRNA-26* promoter and the gRNA sequence, 2 × 35SΩ: 2 × *CaMV35S* promoter with the omega enhancer sequence, Pubi4: parsley *ubiquitin 4-2* promoter, AtCas9: Arabidopsis-codon optimized SpCas9, 2 A: 2 A self-cleavage peptide, Km: the kanamycin resistant marker expression cassette, RB: right border of T-DNA, LB: left border of T-DNA. (**b**) Target sites for *SlIAA9*. 17 b/18 b-length target sequences are in red. The 2–3 base extensions of the 5′ end for the 20 b-length target sequences are in green. The PAM is in blue. A bent arrow indicates the translational start site. (**c**) Visual GFP-selection of transgenic calli with introduced gRNA and Cas9-2A-GFP. Tissues indicated by white circles (white dotted-circles in lower panels “GFP”) were used for mutation analysis or plantlet-regeneration. (**d**) Heteroduplex mobility assay with the MultiNA electrophoresis system. Multiple heteroduplex peaks (red arrows) were detected in PCR amplicons from the CRISPR/Cas9 transgenic tomato calli, whereas a single peak was detected from the wild-type control (blue arrow). VC; vector control. M; internal marker. (**e**) PCR-RFLP analysis of the genome editing in Micro-Tom calli (RNA2-20b). +; *Acc* I digested PCR products, −; non-digested PCR products. Numbers show the regenerated T0 plant lines. (**f**) Determination of mutation ratios in transgenic calli and shoots. The mutation ratios were calculated by dividing number of mutant calli or shoots by the total number of transformed calli or shoots.

To express gRNA targeted to *SlIAA9* in Micro-Tom and Ailsa Craig (see Supplementary Fig. 1) in tomato plants, we constructed two new CRISPR/Cas9 vectors with different promoters for the expression of Cas9 (Fig. 1a). In both vectors, the Arabidopsis *U6-26* promoter was used to express the gRNA constitutively, and either the 2 × *CaMV35S* promoter or parsley *Ubi4-2* promoter was introduced to express an Arabidopsis codon-optimized Cas9 (AtCas9). Both these two vectors differ in codon usage, number of NLSs (three), and the type of *U6* promoter used compared to the former vector pEgP526-2A-GFBSD2^26^ (Supplementary Fig. 2). We designed three gRNAs (gRNA1, gRNA2, and gRNA3) with a target sequence within the second exon of the *SlIAA9*^8^ gene in the tomato genome (Fig. 1b) to introduce mutations in the *SlIAA9* gene using the CRISPR/Cas9 system. Joung’s group reported that 17 bp is the minimum size for a gRNA target sequence in this system (excluding the PAM sequence)^27^, and also showed that, whereas a 20-bp gRNA has a strong capacity to bind to genomic DNA with mismatches in non-target sequences (leading to off-target effects), 17- and 18-bp gRNAs [truncated-gRNA (tru-gRNA)] have high specificity for the target sequence with minimal cleavage activity^27^. Accordingly, we designed a 20-bp gRNA and a17–18 bp tru-gRNA for the target mutagenesis of *SlIAA9*. Target sequences were selected *in silico* using the gRNA design website “focas”^26^. In this website, the CasOT algorithm is used to find potential off-target sites, suggesting an off-target score, and an “on_target_score_calculator.py” algorithm is used to evaluate on-target activity^28^. Using these tools, we selected target sequences that scored high on on-target activity and low on off-target activity. In total, we selected five gRNAs in three sites (Fig. 1b) and inserted these into our CRISPR/Cas9 vectors, and the vectors were used to transform Micro-Tom. Transformed calli were selected using GFP fluorescence (Fig. 1c) and resistance to kanamycin. Transformed calli and shoots with strong GFP fluorescence and good cell growth were then analyzed for the CRISPR/Cas9-induced mutation (Supplementary Fig. 9).

### Detection of CRISPR/Cas9-induced mutations in tomato

To detect mutations in the CRISPR/Cas9 transgenic tomato plants, we used HMA (heteroduplex mobility analysis) (Fig. 1d), PCR-RFLP (restriction fragment length polymorphism), (Fig. 1e and Supplementary Fig. 3), or Cel1-assay (data not shown) of a PCR amplification product of the *SlIAA9* sequence including the target sequences (Supplementary Fig. 9). A heteroduplex of mutant and wild type DNAs from the CRISPR/Cas9 transgenic Micro-Tom calli was detected in HMA as shifted peaks following electrophoresis (Fig. 1d). To determine the mutation rates in the transgenic tomato lines, we performed PCR-RFLP by digestion of the PCR product with the restriction enzyme *Acc* I, looking for uncleaved bands indicating the presence of mutated sequences (Fig. 1e). The representative results of PCR-RFLP analysis of Micro-Tom calli showed that somatic cell level mutation was detected in almost 100% of the transgenic calli induced by gRNA2 (20 bp) using both pEgP237-2A-GFP and pEgPubi4_237-2A-GFP vectors (e.g., tomato lines #4, #5, and #7 with pEgPubi4_237-2A-GFP vector, and #7 and #8 with pEgP237-2A-GFP) (Fig. 1e).

Figure 1f shows the mutation frequencies indicated as the rates of mutated lines to those of CRISPR/Cas9 transgenic tomato lines analyzed by PCR-RFLP (as shown in Fig. 1e). gRNA1 did not induce the mutation in either calli or shoots (data not shown). The mutation rates in Micro-Tom calli were 11.1% for gRNA3 (17 bp) and 40.0–46.2% for gRNA2 (18 bp, 20 bp) and gRNA3 (19 bp) in the pEgP526-2A-GFBSD2 vector^26^ (Supplementary Fig. 2). On the other hand, for gRNA2 (18 bp and 20 bp) and gRNA3 (17 bp), the mutation frequency was 66.7–77.8% in the pEgP237-2A-GFP and pEgPubi4_237-2A-GFP vectors. In Micro-Tom shoots, the highest mutation rates (100%) were detected using gRNA2 (20 bp) in both vectors (Fig. 1f). Thus, pEgP237-2A-GFP and pEgPubi4_237-2A-GFP vectors both enabled the introduction of high-efficiency mutations in tomato. gRNA2 (20 bp) induced the highest mutation efficiency of all the gRNAs tested at the somatic cell level, possibly representing bi-allelic mutation. gRNA2 (20 bp) was also transferred into Ailsa Craig that have the same target sequences in the *SlIAA9* genome (Supplementary Fig. 1).

### Analysis of mutation pattern and off-target mutations

**Figure 2:**
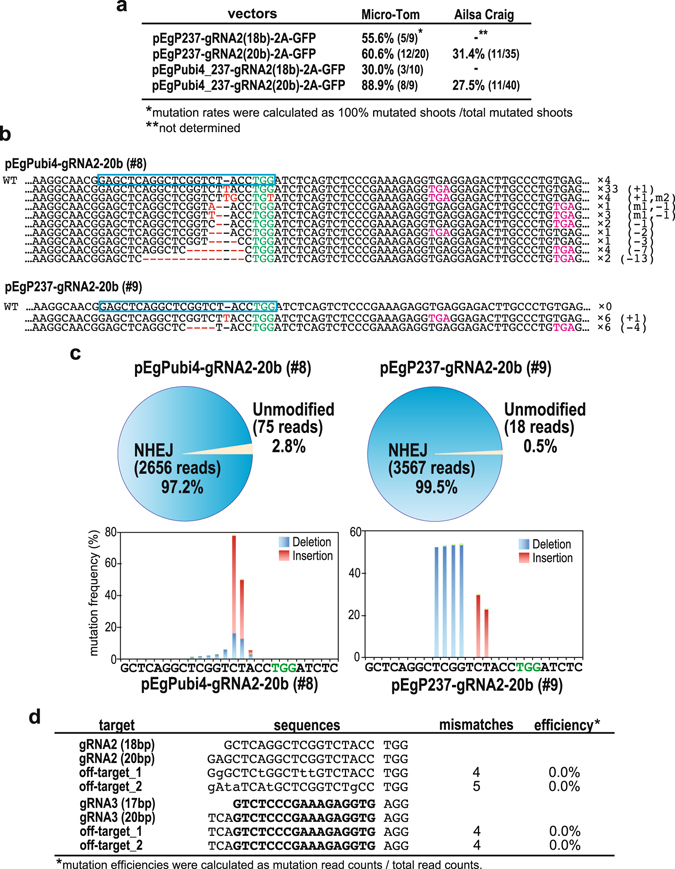
CRISPR/Cas9-induced *SlIAA9* mutations in transgenic tomato calli and shoots. (**a**) Comparison of the rates of high-efficiency mutations (100% mutation at somatic levels detected by PCR-RFLP) using different promoters for Cas9 expression, or different lengths of gRNAs. The mutation rates were calculated by dividing number of 100% mutation shoots by the total number of all-types of mutated shoots. (**b**) Mutation sequences in transgenic calli transformed with pEgPubi-gRNA2-20b (line #8 in Fig. 2) or pEgP237-gRNA2-20b (line #9 in Fig. 2). The WT sequences are shown on top. gRNA target sequences are indicated in blue boxes. Red; mutations generated by CRISPR/Cas9, Magenta; stop codons generated by the CRISPR/Cas9-induced mutations. (**c**) Summary of mutation rates analyzed by NGS in *SlIAA9-crispr* plants. The mutation rates and patterns around the PAM sequence were shown in circle and bar graphs, respectively. Mutation rates were calculated using total read numbers at sequence position. NHEJ; non-homologous end joining. PAM; green nucleotides. (**d**) Mutation rates of off-target sites of gRNA2. Off-target candidates were analyzed by the “focas” website.

PCR-RFLP detected a mutation rate of almost 100% at the somatic cell level induced by gRNA2 (20 bp). We next followed the mutation rates of these high-efficiency mutated shoots in all the regenerated transgenic tomato plants with mutations, and found a high level of mutation (30–90%) in T0 plants of both Micro-Tom and Ailsa Craig (Fig. 2a). To study these mutations in more detail, we then analyzed the mutated sequences in Micro-Tom using Sanger’s method and next-generation sequencing (NGS) by Mi-seq (Fig. 2b,c). We analyzed the sequence of PCR products of 30–60 clones by the Sanger method. From analysis of the sequences derived from shoots of pEgPubi4-gRNA2-20b line #8, which had shown a mutation rate of almost 100% at the somatic level detected by PCR-RFLP (Fig. 1e), we found a plurality of base substitutions, intercalations, deletions, and long stretches of base deletions upstream of the PAM sequence in the cloned DNA (Fig. 2b). In contrast, upon analysis of the cloned DNAs, pEgP237-gRNA2-20b line #9 (Fig. 1e) showed only two types of mutation, at similar levels, suggesting the presence of bi-allelic mutation (Fig. 2b). Using Mi-seq, the amplicon sequence of the mature leaf of pEgPubi4-237-gRNA2-20b line #8 showed that the mutation rate at the somatic cell level was 97.2%, with base insertions and deletions detected 4–5 bp upstream of the PAM sequence (Fig. 2b,c). We also analyzed a mature leaf from tomato line pEgP237-gRNA2-20b #9, and showed that the mutation rate at the somatic cell level was 99.5% (Fig. 2b,c). Base insertion and deletions were detected upstream of the PAM sequence in both transgenic plants (Fig. 2b,c). These data suggest that both our CRISPR/Cas9 vectors can effectively induce mutation with high frequency (near 100%) in regenerated T0 plants in tomato.

To compare the off-target effects obtained with the tru-gRNA compared to the normal 20 bp gRNA, we selected two sequences that have a high risk of off-target effects for each gRNA2. We used Mi-seq to analyze the mutation rate at the somatic cell level at both on- and off-target sites. gRNA2 off-target_1, with 4 mismatches to the gRNA2 on-target sequence located on chromosome 9 (chr9: 68093279–68093302), and gRNA2 off-target_2, with 5 mismatches to the on-target site (chr6: 26946923–26946946), were analyzed (Fig. 2d). The results revealed that no off-target mutations were detected at any of the off-target sites.

### CRISPR/Cas9 *SlIAA9* knockout phenotypes

**Figure 3:**
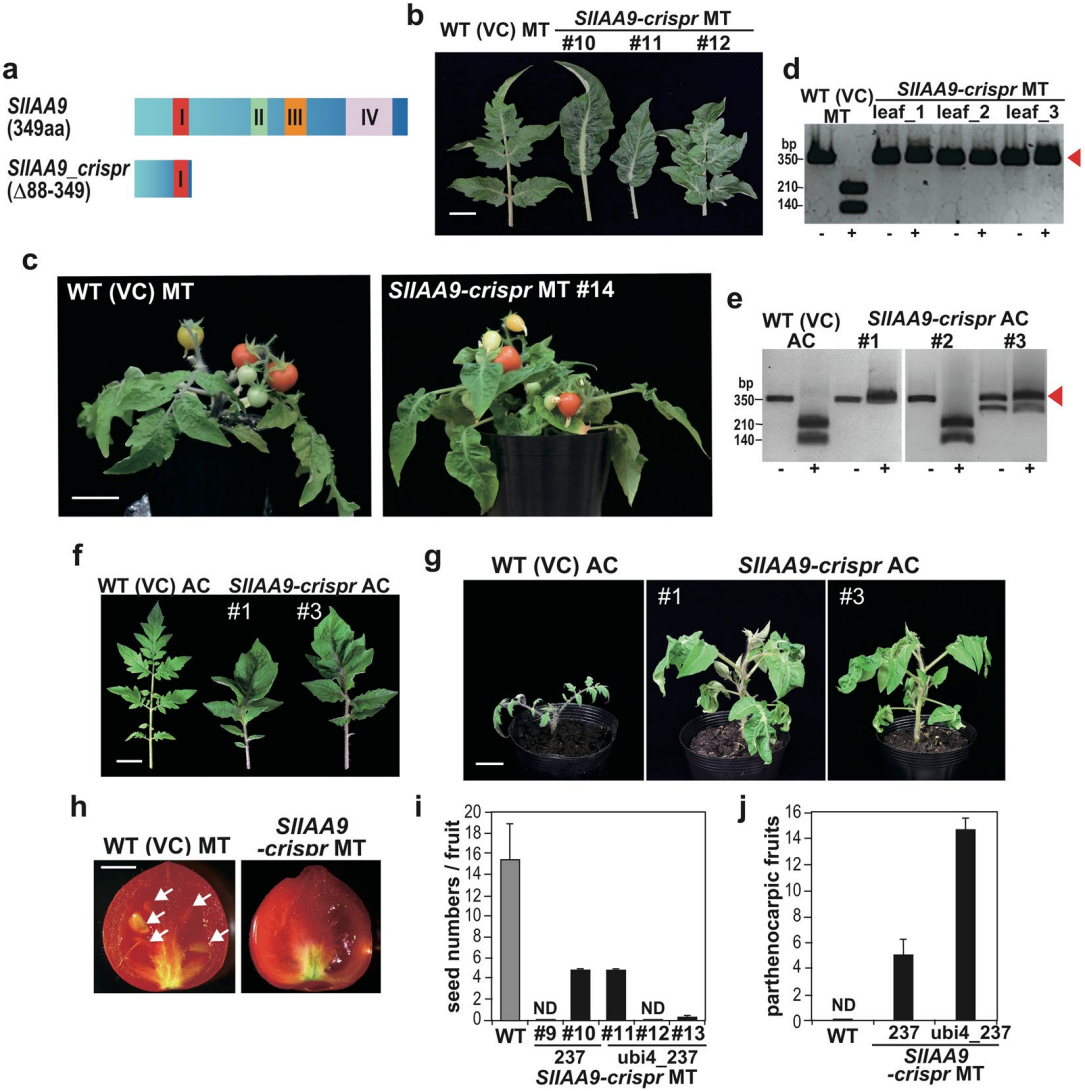
CRISPR/Cas9-induced *SlIAA9* mutations in transgenic tomato plants and their parthenocarpic phenotypes. (**a**) Putative mutant form of the SlIAA9 protein produced by the CRISPR/Cas9 gRNA2. The conserved domains I, II, III and IV of AUX/IAA proteins are indicated. (**b**) Simple leaf morphology in the *SlIAA9-crispr* Micro-Tom plants (#10 and #11 were 100% in PCR-RFLP, #12 showed mild mutation; data not shown). bar = 2 cm. (**c**) Regenerated transgenic T0 plants with fruit formation. bar = 3 cm. (**d**) PCR-RFLP analysis of leaves from pEgPubi4_237-gRNA2-20b T0 #8 (see Supplementary Fig. 5a) (**e**) PCR-RFLP analysis of Ailsa Craig mutants (pEgPubi4_237-gRNA2-20b T0) shown in panel (f). (**f**) Abnormal leaf morphology in the *SlIAA9-crispr* Ailsa Craig plants. bar = 2 cm. (**g**) The *SlIAA9-crispr* Ailsa Craig plants. bar = 3 cm. (**h**) Seedless fruit formation in the *SlIAA9-crispr* T0 Micro-Tom. Seeds in the WT fruit are indicated by white arrows. bar = 5 mm. (**i**) Seed formation rates in *SlIAA9-crispr* Micro-Tom plants. The average seed numbers were calculated in fruits (N = 5–18) in the individual plant lines. (**j**) Fruit formation showing parthenocarpy in *SlIAA9*-*crispr* Micro-Tom plants. Three plants of each construct and WT were used for the measurements. + ; *Acc* I digested PCR products, −; non-digested PCR products. Error bars indicate SE. ND; not detected.

AUX/IAA proteins generally contain four conserved domains (I, II, III, and IV) (Fig. 3a): a repression domain I; domain II, which interacts with auxin receptor TIR1 to control protein stability; and domains III and IV, which play a role in dimerization with Aux/IAA or ARF^29^. A single insertion or 4-base deletion in Micro-Tom (pEgP237-gRNA2-20b T0 #9), and a +1 insertion (pEgPubi4_237-gRNA2-20b T0 #1), or −1 and −73 deletion (pEgPubi4_237-gRNA2-20b T0 #3) in Ailsa Craig, generated a stop codon just after domain I (Fig. 2b and Supplementary Fig. 4). The Ailsa Craig mutants pEgPubi4-gRNA2-20b #1 and #3 have 100% identical mutated sequences, and 50% mutated sequences (bi-allelic), respectively (Supplementary Fig. 4). These mutants include mosaic mutants, e.g., pEgPubi4_237-gRNA2-20b T0 #8 in Micro-Tom (Fig. 2b) disrupted C-terminal domains II, III, and IV, suggesting they would be deficient in functional IAA9.

*SlIAA9* knockout and knockdown have been associated with fruit development prior to pollination, or parthenocarpy, and abnormal leaf morphology (typically exhibiting a simple leaf)^8^. Simple leaves were observed in Micro-Tom CRISPR/Cas9 mutant lines of both pEgPubi4-gRNA2-20b and pEgP237-gRNA2-20b plants (Fig. 3b,c and Supplementary Fig. 5a). Several leaf samples were isolated randomly from mutant plants of pEgPubi4_237-gRNA2-20b T0 #8 and used for analysis of mutation efficiency by PCR-RFLP. The results showed a mutation rate of almost 100% at the somatic cell level in each leaf sample (Fig. 3d). Similarly, in other individual plants with simple leaves, mutated sequences were present at almost 100% at the somatic cell level (data not shown). The CRISPR/Cas9 mutants of Ailsa Craig (Fig. 3e and Supplementary Fig. 4a) also showed altered leaf morphology (Fig. 3f) and likely increased plant growth rates by observation of plants (Fig. 3g).

We further analyzed fruit development and parthenocarpy in the Micro-Tom *SlIAA9*-*crispr* mutants (Fig. 3h–j). Accelerated enlargement of the fruit without self-pollination has been suggested to enhance the development of seedless fruits, and such parthenocarpic characters have been reported in other *SlIAA9* mutants^8^. Seedless fruits were also observed in Micro-Tom *SlIAA9*-*crispr* mutants, whereas wild-type fruits set seed adequately (Fig. 3h,i). The parthenocarpic fruits were detected in the mutants, whereas no parthenocarpic fruits were observed in the wild type (Fig. 3j). Fruit morphology of the mutants was otherwise similar to that of wild-type tomato (Fig. 3c). To obtain successive generations of *SlIAA9*-*crispr* mutants effectively, artificial crossing of the *SlIAA9* mutant and wild-type is an important process in yielding the seedless phenotype (Supplementary Fig. 5b). We then analyzed the mutation efficiency in pollen, representing the haploid male gametophyte of Micro-Tom *SlIAA9*-*crispr* mutants, by PCR-RFLP. The results indicated a high mutation efficiency, suggesting that efficiency is sufficiently high to generate heterozygotes in the next generation (Supplementary Fig. 5c–e). Furthermore, in the few collected seeds of *SlIAA9*-*crispr* mutants, a high mutation frequency was also detected (T1 seeds; Supplementary Fig. 6), and next generation plants showed the typical simple leaf phenotype upon germination (T1 seeds; Supplementary Fig. 6b). We further analyzed the fruit phenotypes and the expression levels of several marker genes in the mutants, and showed that the development of mutant fruits was similar to that of wild-type (Supplementary Fig. 7). Together, these results suggest that our highly efficient CRISPR/Cas9 system for tomato was successful in generating almost 100% mutagenesis in the T0 generation, and of establishing heritable mutation effectively to obtain mutant tomato lines in successive generations.

## Discussion

Current advances in new breeding techniques such as genome editing are now available also in crop plants. Here, we developed highly efficient genome editing using the CRISPR/Cas9 system in tomato, demonstrating for the first time the feasibility of this genome editing-based breeding technique to produce parthenocarpic tomato plants by disruption of the *SlIAA9* gene. Wang *et al.*^8^ reported that the *SlIAA9* gene is expressed throughout leaf and fruit ontogeny (Supplementary Fig. 8) and that downregulation of the *IAA9* gene using antisense RNA caused parthenocarpy, leading them to suggest that the IAA9 protein prevents ovary development prior to pollination in wild-type tomato. In this study, we optimized the CRISPR/Cas9 system in tomato in terms of the promoters used for gRNA and Cas9 expression, the number of NLS in Cas9, and the length of gRNA target sequence, and established a set of expression cassettes yielding highly efficient site-directed mutagenesis on the specified target in both a model tomato and the commercial cultivar Ailsa Craig (Figs 1 and 3). Our work has shown that several optimizations of the basal cassettes in CRISPR/Cas9 enhanced the mutation efficiency in both tomato cultivars dramatically. While it is difficult to ascribe the improvement to a specific or multiple elements in the vectors from our current data, the enhancement can be attributed to the new vectors as a whole. Our results suggest that the CRISPR/Cas9 system is indeed one of the strongest tools available for gene editing to date, with more potential for further improvements in this effective system.

In transgenic plants regenerated from callus propagated on excised leaf disks, both gRNA and Cas9 expression need to be maintained at high levels during callus formation and propagation to ensure efficient mutagenesis. The off-target effects of the CRISPR/Cas9 system have been studied extensively, and it was found that the tolerance of the gRNA to multiple mismatches within the protospacer sequence also induced DSBs in some non-target genomic sequences^30–33^. Regeneration and micropropagation are important techniques for breeding of some useful crops, such as tree species whose seeds are difficult to produce. In such cases, a high frequency of off-target effects at the somatic level that would cause undesired mutations in the regenerated plants is one of the high risks during the generation of individual plants with newly induced on-target mutations. Therefore, the reduction of off-target effects is highly desired, especially in crop species. In this study, we designed highly specific gRNAs aimed at avoiding off-target effects; one such strategy was to use truncated gRNA (tru-gRNA) targets with 17- to 18-b target sequences. tru-gRNAs have been used in mammalian and Arabidopsis cells, and indeed have been shown to reduce the risk of off-target effects^26^. In tomato, our tru-gRNA targeting *SlIAA9* introduced on-target mutations efficiently with no off-targets as judged by amplicon sequence analysis (Fig. 2). Interestingly, the 20 b gRNA2 target sequence in *SlIAA9* also led to high on-target mutation with no off-target effects, suggesting that we had selected highly specific gRNAs. Further application of *in silico* tools, such as the on-target score calculator^26,28^, and improvements of the vectors, will continue to contribute the development of efficient genome editing systems without off-target mutations in various useful crop species.

We obtained bi-allelic and homozygous mutations in T0 regenerated plants of Micro-Tom as well as in the finest cultivar Ailsa Craig. Using this system, T0 plants with the desired phenotypes and 100% mutation in the target gene in general cultivars were established with high efficiency (Fig. 2). Mosaic mutations were also found in these cultivars; however, several lines exhibited almost 100% mutated target sequences at the somatic level (Fig. 2) in individual mutant plants with strong phenotypes, since no normal IAA9 protein molecules were generated in the knock-out mutants. The *SlIAA9* CRISPR/Cas9 knock-out tomato exhibited the typical phenotypes of parthenocarpy, which processes fruiting without fertilization and leads to seedless fruits. In *SlIAA9-crispr*, seedless tomato fruits were generated; however, in a very few cases, small numbers of fertilized fruits developed a few seeds, which grew with phenotypes exhibiting the heritable mutation (Supplementary Fig. 6). In vegetative tissues, *SlIAA9-crispr* exhibited the leaf phenotype typical of other *SlIAA9* mutants generated by chemical, physical, or RNAi induced mutation^8^. These abnormal leaf phenotypes do not negatively affect plant growth. New developments in delivery systems for CRISPR-Cas9 in plant cells^34,35^ using our efficient system would also advance the further application of parthenocarpy and other important traits, such as stress tolerance, in various useful crops in future.

## Methods

### Plant material, growth conditions, and transformation

Tomato plants (*Solanum lycopersicum L.*) cv. Micro-Tom and Ailsa Craig were grown in a growth chamber under conditions of 21–25 °C with 16 h light at 4000–6000 lx/8 h dark. Transgenic tomato plants were generated using the CRISPR/Cas9 vectors by the *Agrobacterium*-mediated leaf disk method (Supplementary Fig. 9). Briefly, cut cotyledon pieces (7–10 days after seeding) were transformed with *A. tumefaciens* strain GV2260 harboring the CRISPR/Cas9 plasmid. Transgenic calli were selected on MS medium containing 0.1 μg/mL blasticidin S or 100 μg/mL kanamycin, and by observing GFP fluorescence. Around 20–40 leaf disks transformed with CRISPR/Cas9 were cultured in each individual experiment. The three independent experiments were performed. We finally regenerated around 40 CRISPR/Cas9 mutant plants that showed similar parthenocarpic phenotypes.

### Design of gRNA and CRISPR/Cas9 vectors

To design target gene sequences with low off-target effects in the second exon of *SlIAA9* genome DNA (*Solyc04g076850*) (Supplementary Fig. 1 and Supplementary Fig. 8), we used the web-tool “focas” (http://focas.ayanel.com) (Osakabe *et al*.^26^) and analysis using Cas-OT software (Xiao *et al*., 2014). Three candidate sequences of tru-gRNA targets (17–18 bp) were selected and named gRNA1-17b, gRNA2-18b, and gRNA3-17b. An additional 2 bp was added to each tru-gRNA to target 20 bp sequences at the sites for gRNA2 and gRNA3; these additional gRNAs were named gRNA2-20b and gRNA3-20b. At the 5′-end of gRNA3-20b, a G that does not exist in the wild-type genome was added to its 19-bp sequence to allow efficient expression from the U6 promoter (Fig. 1c). Stop codons that would be generated by a frame shift were located downstream of each target sequence. The annealed oligos for gRNAs were transferred into CRISPR/Cas9 vectors using Golden Gate Cloning methods. In pEgP526-2A-GFBSD2^26^, a gRNA cassette harboring the Arabidopsis *U6 snRNA-1* (*U6-1*) promoter, the fungal and plant codon-optimized *Streptococcus pyogenes Cas9* (*fcoCas9*), and *GFBSD2* was placed under the control of the *2* × *CaMV35S* promoter. *GFBSD2* was fused to *fcoCas9* via a self-cleaving 2A peptide derived from *Thosea asigna*, to monitor the GFP fluorescence used to select Cas9-expressing plant cells. In pEgP237-2A-GFP, gRNA under the Arabidopsis *U6 snRNA-26* (*U6-26*) promoter, a Arabidopsis codon-optimized *S. pyogenes Cas9* (*AtCas9*) and *GFP* were driven by the *2* × *CaMV35S* promoter. For pEgPubi4_237-2A-GFP, the *U6-26* promoter was used for gRNA expression, and a parsley *ubi4-2* promoter was used for Cas9 expression.

### Detection of CRISPR/Cas9-induced mutation

To detect mutations in the target sequences, genomic DNA was isolated from the transformed calli, regenerated shoots, leaf tissues, fruits, and pollen of plants selected by GFP fluorescence and chemical tolerance. To evaluate mutations introduced in the CRISPR/Cas9 transgenic plants, a region of about 300 bp surrounding the target locus of gRNA was amplified by PCR. To detect mutations in the PCR product, HMA using MCE202 MultiNA (Shimadzu, Japan), Cel-1 assay, or PCR-RFLP was first used. In HMA, the PCR fragments were analyzed directly using a microchip electrophoresis system with MCE202 MultiNA. In the Cel-1 assay, the PCR products for gRNA1 and gRNA3 were digested with Guide-it™ Mutation Detection Kit (Takara, Japan) or Surveyor® Mutation Detection Kits (Transgenomic, USA). In PCR-RFLP, the PCR products for gRNA2 were digested with *Acc* I. The mutated and the wild-type DNA fragments were separated by agarose-gel electrophoresis.

### Sequencing analyses

In Sanger’s sequencing method, the PCR product was cloned into a plasmid T-Vector pMD 20 (Takara, Japan). The resulting plasmids were used to transform *E. coli* strain DH5α, about 30–50 white colonies were selected from which to extract and sequence the plasmid DNA containing the mutation. In next-generation sequencing by Mi-seq (illumina, Japan), genomic DNA including the mutation was used to amplify the region including gRNA2 and gRNA3 target sequences by PCR. Similarly, two selected sequences with high risk of off-target effects from off-target candidate sequences of gRNA2 (chr9: 68093279–68093302 and chr6: 26946923–26946946) or gRNA3 (chr12: 23706808–23706831 and chr3: 63407250–63407273) selected by Cas-OT were examined. First PCR products were extracted from the gel using the Wizard® SV Gel and PCR Clean-Up System (Promega, Japan) and used as templates for a second round of PCR. Second PCR primers were subjected to Truseq (illumina, Japan). Mi-seq data (about 11000–30000 reads) was analyzed using CLC Genomics Workbench software version 7.5.1 (CLC bio, Japan), mapped on the *SlIAA9* sequence using Integrative Genomics Viewer (IGV) (Broad Institute) and graphing of deletion, insertion and substitution on the target sequence was generated using Microsoft Excel and CRISPResso (http://crispresso.rocks).

### Phenotypical characterization of CRISPR/Cas9-induced *SlIAA9* mutant

Plant phenotypes (leaves and fruits) in the CRISPR/Cas9 *SlIAA9* mutants with highly mutated sequences were evaluated. To evaluate tomato parthenocarpy, seed numbers and formation were measured in the cut fruits. The average seed numbers was calculated in 5–18 fruits from the individual plant lines. Number of the parthenocarpic fruits in three plants of each construct and WT was counted and the average numbers were calculated.

### qRT-PCR analysis

Total RNA was extracted using RNAiso Plus (Takara, Japan) from buds, flowers, mature green fruits, and red fruits. Reverse transcription was performed using the purified RNA with SuperScript III and Random hexamer primer (Invitrogen, Carlsbad, USA). qRT-PCR was performed in a LightCycler system (Roche Molecular Biochemicals, Germany) using SYBR Premix Ex Taq (Takara, Japan). The qRT-PCR primers are listed in Supplementary Table 1. Other experimental conditions were as previously described^36,37^.

## Electronic supplementary material

Supplementary table and figure
