# Author Correction: Rapid breeding of parthenocarpic tomato plants using CRISPR/Cas9

**DOI:** 10.1038/s41598-020-71765-6

**Published:** 2020-10-02

**Authors:** Risa Ueta, Chihiro Abe, Takahito Watanabe, Shigeo S. Sugano, Ryosuke Ishihara, Hiroshi Ezura, Yuriko Osakabe, Keishi Osakabe

**Affiliations:** 1 Graduate School of Advanced Technology and Science, Tokushima University, Tokushima, Japan; 2 Center for Collaboration among Agriculture, Industry, and Commerce, Tokushima University, Tokushima, Japan; 3 Graduate School of Life and Environmental Sciences, University of Tsukuba, Tsukuba, Japan; 4 Faculty of Bioscience and Bioindustry, Tokushima University, Tokushima, Japan

Correction to: *Scientific Reports* 10.1038/s41598-017-00501-4, published online 30 March 2017.

In Figure 2D, three of the guide RNA sequences provided are incorrect.

The sequence for the off-target site, ‘off-target_2′, of gRNA2 which was ‘gAtaTCAtGCTCGGTCTgCC’ should read ‘tAtaTCAtGCTCGGTCTgCC’, the sequence for the off-target site, ‘off-target_1′, of gRNA3 which was ‘TCAGTCTCCCGAAAGAGGTG’ should read ‘gCAGcCTtCaGAAAGAGGTG’, and the sequence for the off-target site, ‘off-target_2′, of gRNA3 which was ‘TCAGTCTCCCGAAAGAGGTG’ should read ‘TtAGTCaCtaGAAAGAGGTG’.

The correct Figure 2 appears below as Figure 1.

**Figure 1:**
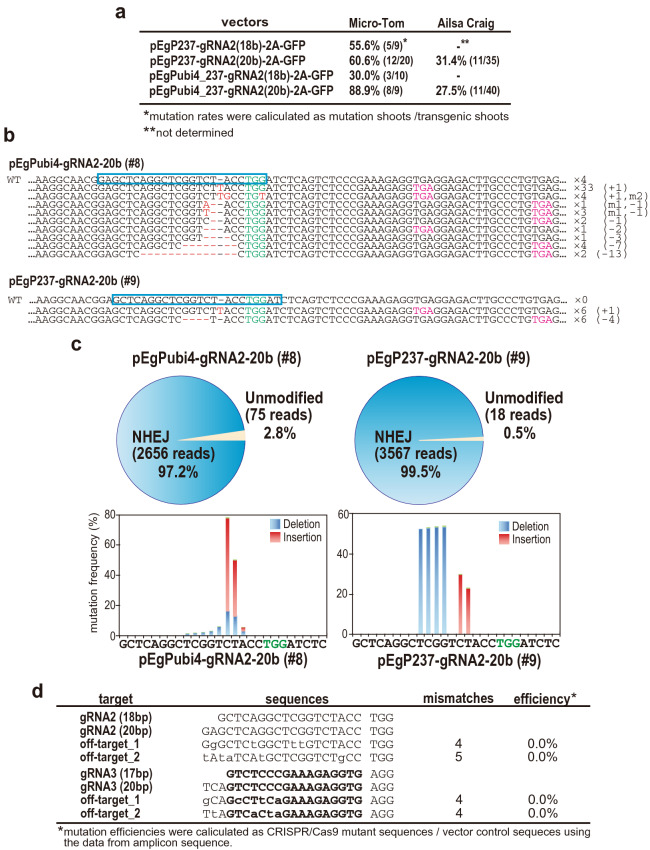
CRISPR/Cas9-induced SlIAA9 mutations in transgenic tomato calli and shoots. (**a**) Comparison of the rates of high-efficiency mutations (100% mutation at somatic levels detected by PCR-RFLP) using different promoters for Cas9 expression, or different lengths of gRNAs. The mutation rates were calculated by dividing number of 100% mutation shoots by the total number of all-types of mutated shoots. (**b**) Mutation sequences in transgenic calli transformed with pEgPubi-gRNA2-20b (line #8 in Fig. 2) or pEgP237-gRNA2-20b (line #9 in Fig. 2). The WT sequences are shown on top. gRNA target sequences are indicated in blue boxes. Red; mutations generated by CRISPR/Cas9, Magenta; stop codons generated by the CRISPR/Cas9-induced mutations. (**c**) Summary of mutation rates analyzed by NGS in SlIAA9-crispr plants. The mutation rates and patterns around the PAM sequence were shown in circle and bar graphs, respectively. Mutation rates were calculated using total read numbers at sequence position. NHEJ; non-homologous end joining. PAM; green nucleotides. (**d**) Mutation rates of off-target sites of gRNA2. Off-target candidates were analyzed by the “focas” website.

