# Supplementary material for: Rapid breeding of parthenocarpic tomato plants using CRISPR/Cas9
Source: Sci Rep. 2017 Mar 30;7:507. doi: 10.1038/s41598-017-00501-4 (PMC5428692; doi:10.1038/s41598-017-00501-4)
Supplement: Supplementary file 1 — Supplementary table and figure [file 41598_2017_501_MOESM1_ESM.pdf]

Title: Rapid breeding of parthenocarpic tomato plants using CRISPR/Cas9

Authors:

Risa Ueta[1], Chihiro Abe[1], Takahito Watanabe[2], Shigeo S Sugano[2], Ryosuke Ishihara[1], Hiroshi Ezura[3], Yuriko Osakabe[4], Keishi Osakabe[4*]

Affiliations:

[1]Graduate School of Advanced Technology and Science, Tokushima University, Japan

[2]Center for Collaboration among Agriculture, Industry, and Commerce, Tokushima University, Japan

[3]Graduate School of Life and Environmental Sciences, University of Tsukuba, Tsukuba, Japan

[4]Faculty of Bioscience and Bioindustry, Tokushima University, Japan

Corresponding Author

Keishi Osakabe

Faculty of Bioscience and Bioindustry, Tokushima University, Japan

Tel: +81-88- 634-6418

kosakabe@tokushima-u.ac.jp

Supplementary Table 1. Primer list for construction of CRISPR/Cas9 vector and PCR for Cel-1 assay, deep sequencing, and qRT-PCR

primers	sequence of primers	
gRNA1-17bF	5' GATTGTGTGGATTAAATCTCA 3'	For constructing the CRISPR/Cas9 vector
gRNA1-17bR	5' AAACTGAGATTTAATCCACAC 3'	For constructing the CRISPR/Cas9 vector
gRNA2-18bF	5' GATTGCTCAGGCTCGGTCTACC 3'	For constructing the CRISPR/Cas9 vector
gRNA2-18bR	5' AAACGGTAGACCGAGCCTGAGC 3'	For constructing the CRISPR/Cas9 vector
gRNA2-20bF	5' GATTGAGCTCAGGCTCGGTCTACC 3'	For constructing the CRISPR/Cas9 vector
gRNA2-20bR	5' AAACGGTAGACCGAGCCTGAGCTC 3'	For constructing the CRISPR/Cas9 vector
gRNA3-17bF	5' GATTGTCTCCCGAAAGAGGTG 3'	For constructing the CRISPR/Cas9 vector
gRNA3-17bR	5' AAACCACCTCTTTCGGGAGAC 3'	For constructing the CRISPR/Cas9 vector
gRNA3-20bF	5' GATTGTCAGTCTCCCGAAAGAGGTG 3'	For constructing the CRISPR/Cas9 vector
gRNA3-20bR	5' AAACCACCTCTTTCGGGAGACTGAC 3'	For constructing the CRISPR/Cas9 vector
iaa9-F27-52	5' GGAGGAGGAGGGCCAGAGTAATGTAA 3'	For Cel1-assay or PCR-RFLP of the target sequence region
iaa9-R375-348	5' GTTGCCACTAACTACTGTTTTCTGCGAT 3'	For Cel1-assay or PCR-RFLP of the target sequence region
F2_IAA9-2	5' ACACTCTTTCCCTACACGACGCTCTTCCGATCTAGGACAATAATGGGTGTGGA 3'	For next-generation sequencing, 1st PCR primer for on-targets, gRNA2 and gRNA3
R2_IAA9-2	3' GTGACTGGAGTTCAGACGTGTGCTCTTCCGATCTCAGCTTCTCATCAACCTTTGT 5'	For next-generation sequencing, 1st PCR primer for on-targets, gRNA2 and gRNA3
F3_18b_IAA9-2_off1	5' ACACTCTTTCCCTACACGACGCTCTTCCGATCTGCTCTCGCTCTTGCTCTCT 3'	For next-generation sequencing, 1st PCR primer for off-target 1 of gRNA2
R3_18b_IAA9-2_off1	3' GTGACTGGAGTTCAGACGTGTGCTCTTCCGATCTACTACAACACGAAATCTACAA 5'	For next-generation sequencing, 1st PCR primer for off-target 1 of gRNA2
F_18b_IAA9-2_off2	5' ACACTCTTTCCCTACACGACGCTCTTCCGATCTGGAAGTTATTCAAACAAGCCAA 3'	For next-generation sequencing, 1st PCR primer for off-target 2 of gRNA2
R_18b_IAA9-2_off2	3' GTGACTGGAGTTCAGACGTGTGCTCTTCCGATCTTTGAGAATCATTCAAGTGGTTA 5'	For next-generation sequencing, 1st PCR primer for off-target 2 of gRNA2
F_17b-3_off1'	5' ACACTCTTTCCCTACACGACGCTCTTCCGATCTGGAGACATTTGGGCACCATT 3'	For next-generation sequencing, 1st PCR primer for off-target 1 of gRNA3
R_17b-3_off1'	3' GTGACTGGAGTTCAGACGTGTGCTCTTCCGATCTAAACTTTAGCCCTTTGAATCA 5'	For next-generation sequencing, 1st PCR primer for off-target 1 of gRNA3
F_17b-3_off2	5' ACACTCTTTCCCTACACGACGCTCTTCCGATCTCCTTCATCCTTCGTCACTGT 3'	For next-generation sequencing, 1st PCR primer for off-target 2 of gRNA3
F_17b-3_off2	3' GTGACTGGAGTTCAGACGTGTGCTCTTCCGATCTGGACTTCTTAGGGAAGCTCAA 5'	For next-generation sequencing, 1st PCR primer for off-target 2 of gRNA3
SlARF17_Fw	5' TGAAGTTGATGAAGTTACTATGAG 3'	For qRT-PCR of ARF17
SlARF17_Rv	5' TCCTCCATTATTCGCATCTG 3'	For qRT-PCR of ARF17
SlARF2A_Fw	5' GCAAGGTCAAGAGTTATCGA 3'	For qRT-PCR of ARF2A
SlARF2A_Rv	5' CATTGGTTTCTCAGACAAGTC 3'	For qRT-PCR of ARF2A
SlASR4_Fw	5' GGTAATGAGGAAGGTGGCTATGG 3'	For qRT-PCR of ASR4
SlASR4_Rv	5' TGGTTCCACTATCATCATTCTCTTCA 3'	For qRT-PCR of ASR4
Sl-Actin-51_Fw	5' TGTCCCTATCTACGAGGGTTATGC 3'	For qRT-PCR of control
Sl-Actin-51_Rv	5' AGTTAAATCACGACCAGCAAGAT 3'	For qRT-PCR of control

***SlIAA9* (MicroTom)**	1>AGCATATGCATAAAAGGATCAGCTCTTAAAGAGCGAAACTATATGGGTCTATCTGATTGTTCGTCGGTGGACAGCTGTAATATTTCCACC>90
***SlIAA9* (Ailsa Craig)**	1>AGCATATGCATAAAAGGATCAGCTCTTAAAGAGCGAAACTATATGGGTCTATCTGATTGTTCGTCGGTGGACAGCTGTAATATTTCCACC>90
***SlIAA9* (MicroTom)**	91>TCATCAGAGGACAATAATGGGTGTGGATTAAATCTCAAGGCAACGGAGCTCAGGCTCGGTCTACC**TGG**ATCTCAGTCTCCCGAAAGAGGT>180
***SlIAA9* (Ailsa Craig)**	91>TCATCAGAGGACAATAATGGGTGTGGATTAAATCTCAAGGCAACGGAGCTCAGGCTCGGTCTACCTGGATCTCAGTCTCCCGAAAGAGGT>180
***SlIAA9* (MicroTom)**	181>GAGGAGACTTGCCCTGTGATTTCGACAAAGGTTGATGAGAAGCTGCTCTTCCCCTTGCACCCTTC>245
***SlIAA9* (Ailsa Craig)**	181>GAGGAGACTTGCCCTGTGATTTCGACAAAGGTTGATGAGAAGCTGCTCTTCCCCTTGCACCCTTC>245

Supplementary Figure 1. The *SlIAA9* sequences in the CRISPR/Cas9 target regions from Micro-Tom and Ailsa Craig cultivars. The identical genome sequences were isolated from the two cultivars.

a

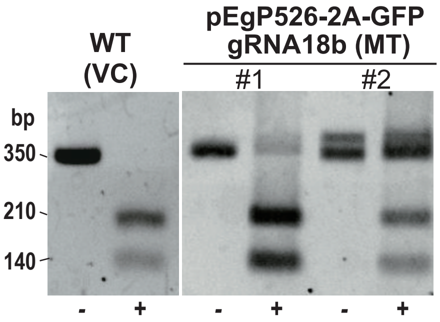

b

vectors	Micro-Tom calli	Micro-Tom shoots
pEgP526-gRNA1(17b)-2A-GFBSD2	0.0% (0/12)*	0.0% (0/27)
pEgP526-gRNA2(18b)-2A-GFBSD2	46.2% (6/13)	33.3% (14/42)
pEgP526-gRNA2(20b)-2A-GFBSD2	45.5% (5/11)	-**
pEgP526-gRNA3(17b)-2A-GFBSD2	11.1% (1/9)	12.5% (2/16)
pEgP526-gRNA3(20b)-2A-GFBSD2	40.0% (4/10)	-

*mutation rates were caliculated as mutation shoots /transgenic shoots
**not determined

Supplementary Figure 2. a. PCR-RFLP analysis of the pEgP526-gRNA2 (18b) transgenic Micro-Tom. +; *Acc* I digested PCR products, -; non-digested PCR products. **b.** Mutation efficiency of Micro-Tom transformed with pEgP526 vectors.

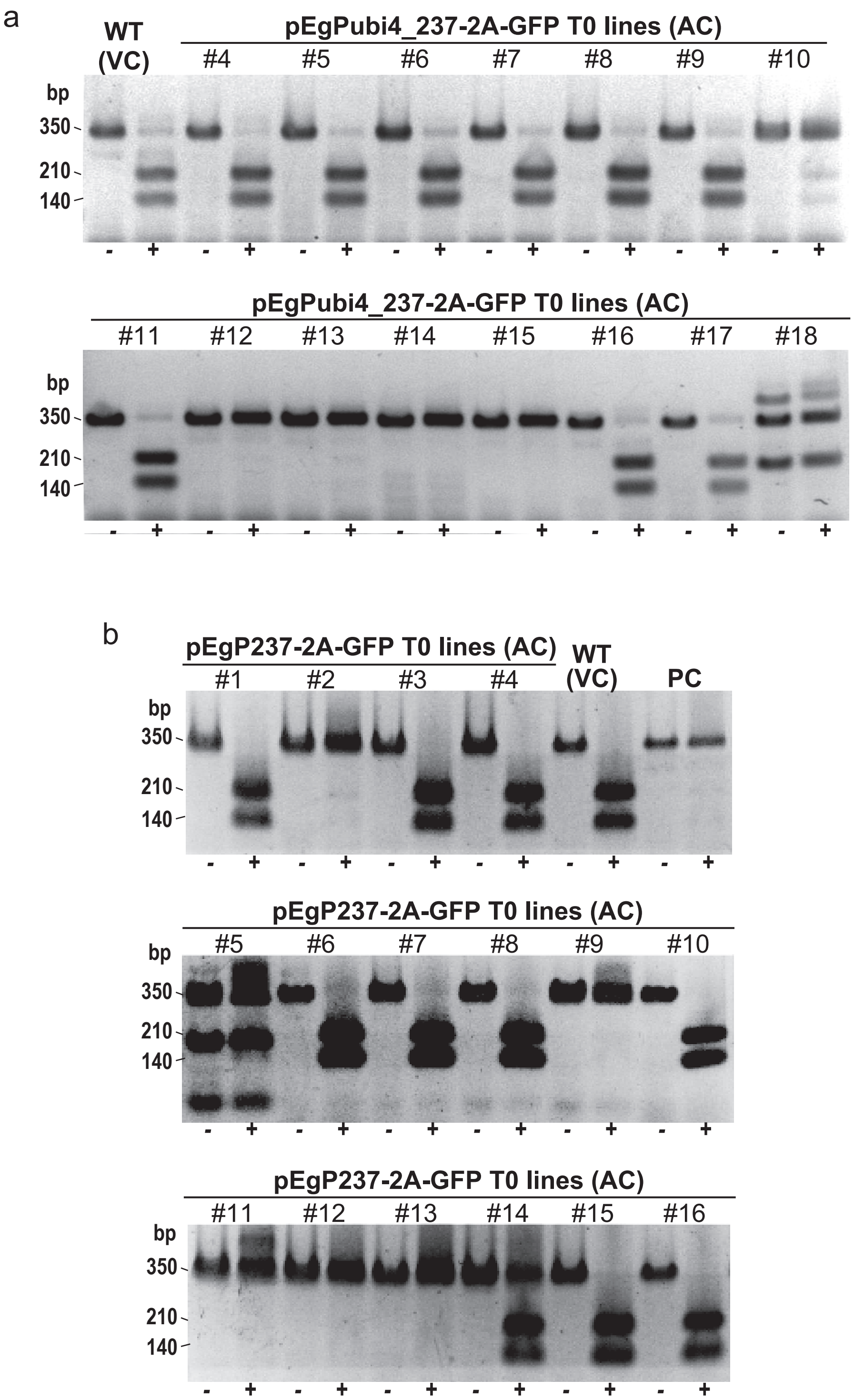

Supplementary Figure 3. PCR-RFLP analysis of the *SlIAA9* gene mutations induced by CRISPR/Cas9 vectors in Ailsa Craig cultivars. +; Acc I digested PCR products, –; non-digested PCR products.

a. pEgPubi4_237-2A-GFP T0 shoots (#4–#18). The data of #1–#3 plants were presented in Fig. 3.

b. pEgP237-2A-GFP T0 shoots (#1–#16). PC; positive control using the pEgPubi4_237-2A-GFP T0 100% mutation line.

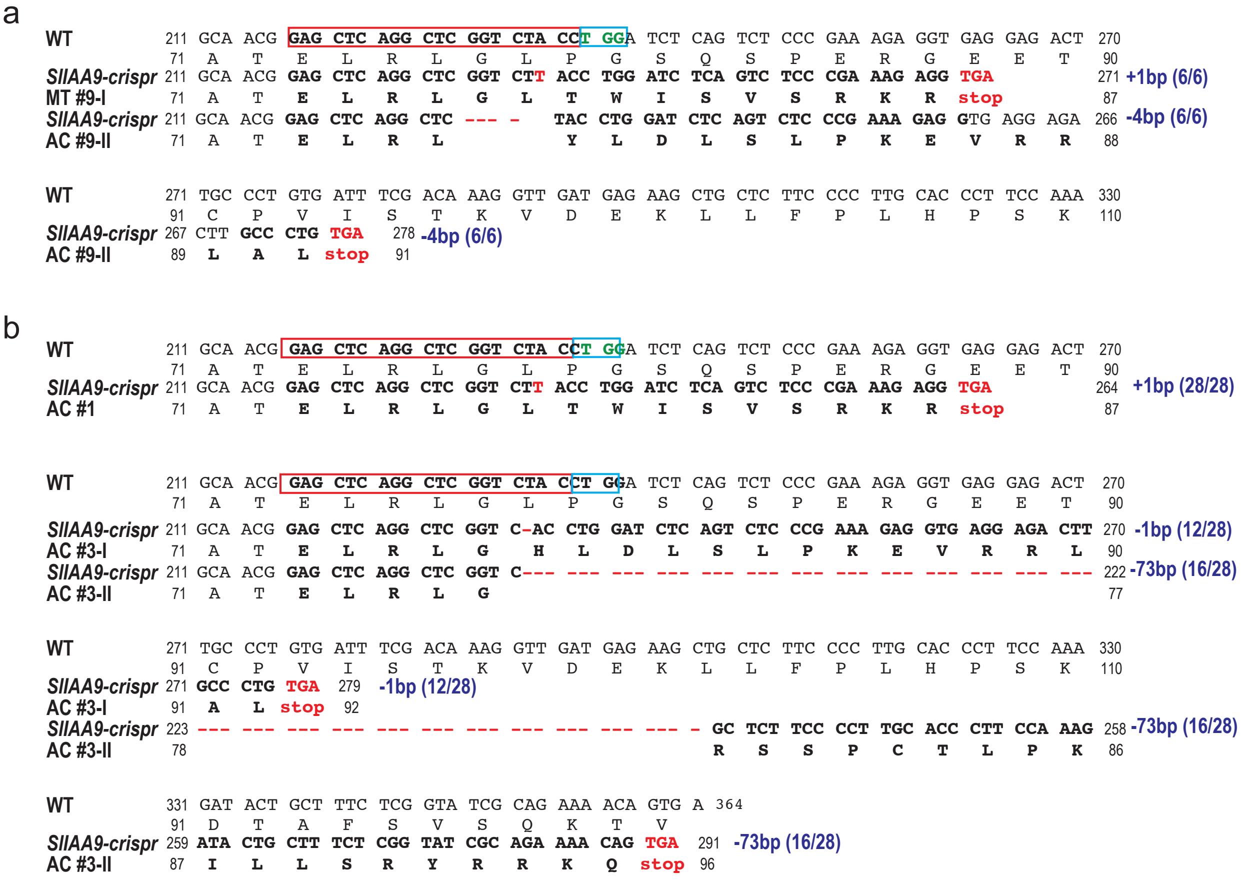

Supplementary Figure 4. *SlIAA9* gene bi-allelic mutations induced by CRISPR/Cas9 vectors in Micro-Tom and Ailsa Craig T0 plants. **a.** SlIAA9 sequences of pEgP237-2A-GFP T0 Micro-Tom (#9). **b.** *SlIAA9* sequences of pEgPubi4_237-2A-GFP Ailsa Craig (#1 and #3).

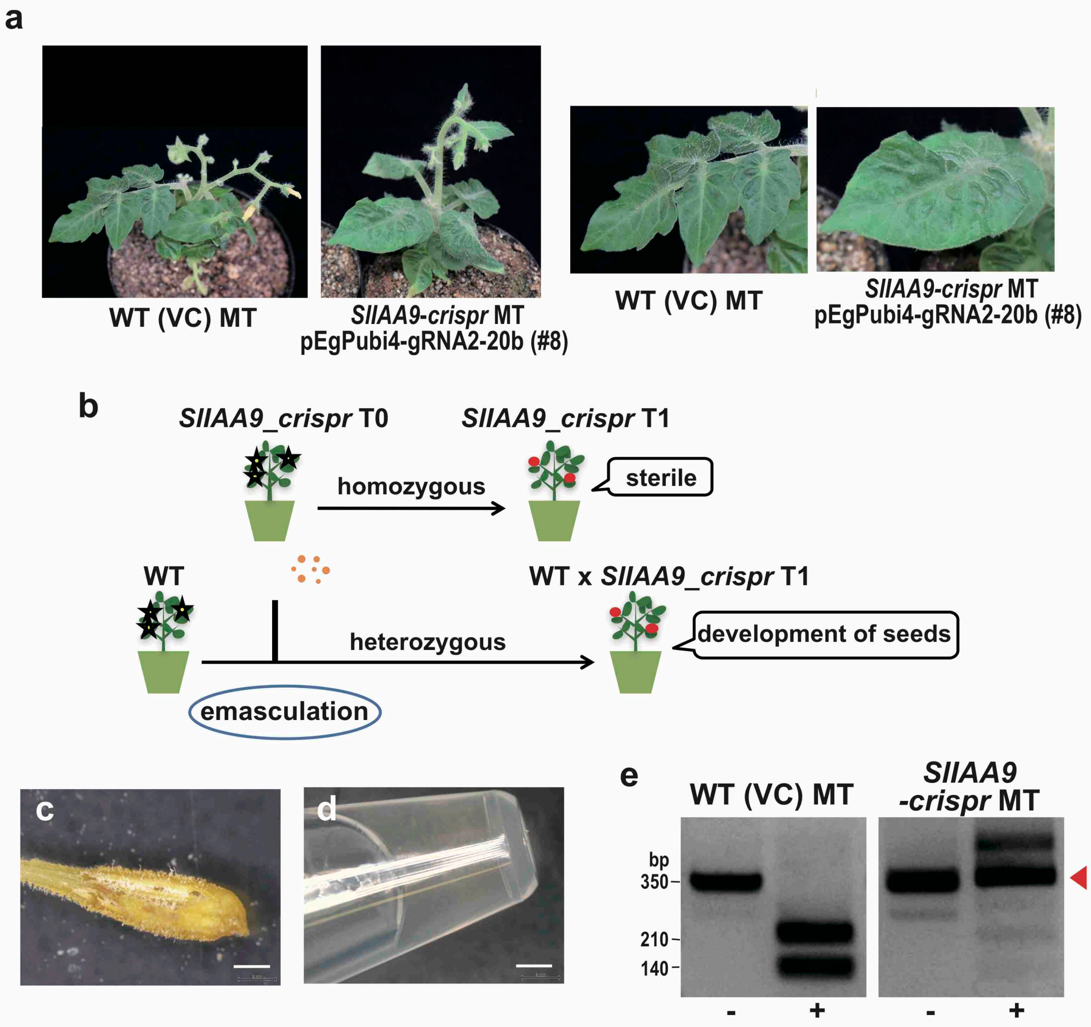

Supplementary Figure 5. a. Leaf morphology of the *SlIAA9-crispr* Micro-Tom (#8) induced by pEgPubi4_237-2A-GFP. **b.** Strategy to propagate *SlIAA9-crispr* mutants with parthenocarpic phenotype by crossing with wildtype and developing the heterozygous T1 generation. **c-e.** PCR-RFLP analysis (e) of Micro-Tom pollen grains (d), which are haploid male gametophytes isolated from the mature flower of the *SlIAA9-crispr* mutant (c). bar = 2 mm. +; *Acc* I digested PCR products, -; non-digested PCR products.

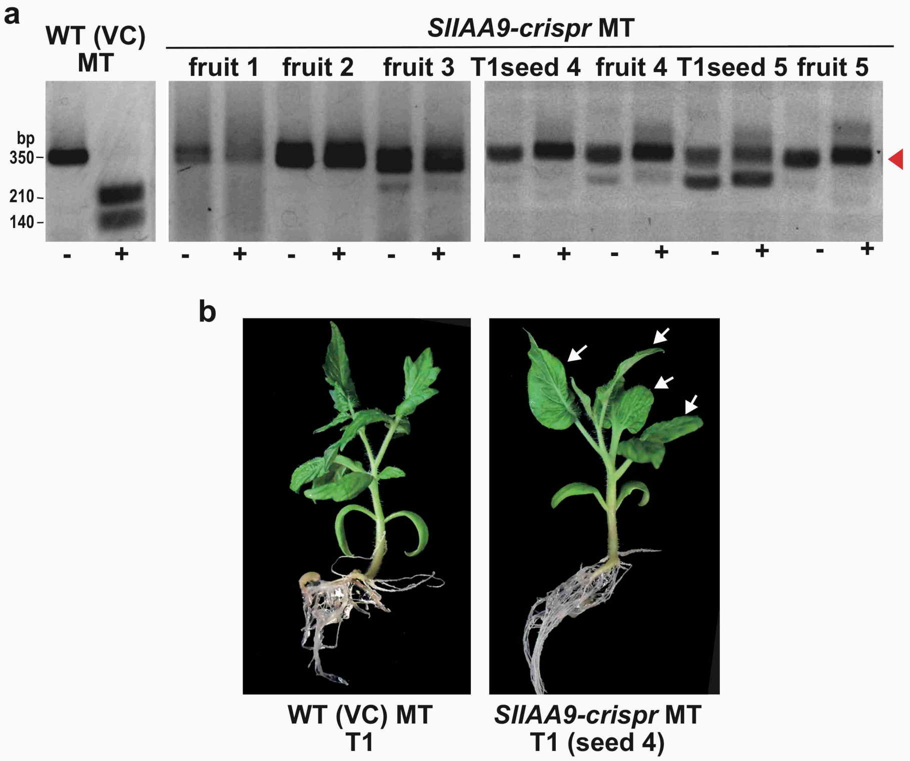

Supplementary Figure 6. Segregated mutation of *SlIAA9-crispr* mutants in the T1 generation. **a.** PCR-RFLP analysis of Micro-Tom fruits (fruits 1 and 2 were pEgP237-2A-GFP #9 and #10, fruits 3, 4, 5 were pEgPubi4_237-2A-GFP#13, #12, #11, respectively) and the T1 seeds generated from several T0 plants at low efficiency. Fruits 1–3 showed non-seed phenotypes. +; *Acc* I digested PCR products, -; non-digested PCR products. **b.** The *SlIAA9-crispr* mutant T1plant generated from seed #4 in a. Arrows show the abnormal leaf morphology (simple leaves).

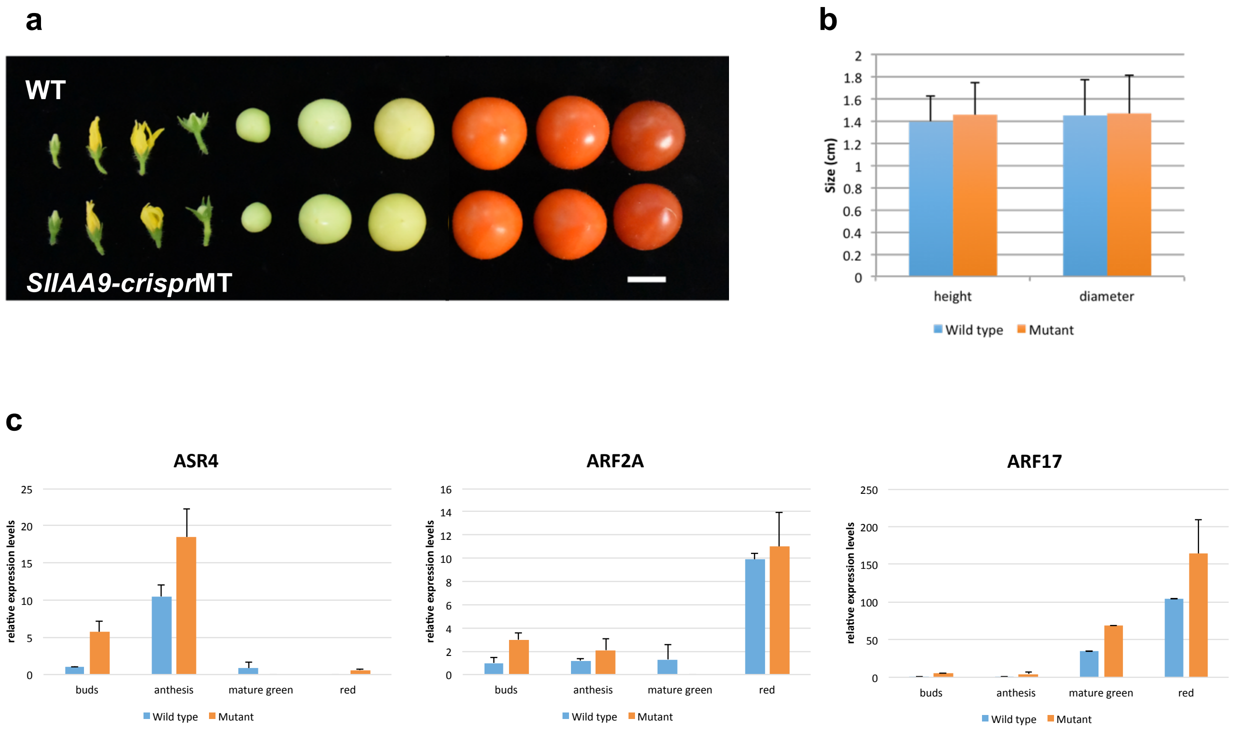

Supplementary Figure 7. a. The tomato fruit development and maturation process; buds, before anthesis, fertilization, immature green fruits, mature green fruits, breaker fruits, orange fruits, red fruits, and over-ripe fruits. *Top row* Wild-type, *lower row SlIAA9-crispr* MT. Scale bar = 1 cm. b. Comparison of height and diameter of wild-type fruits and *SlIAA9-crispr* mutant fruits. Values are means ± SD (Wild type $n = 47$, Mutant $n = 50$). c. Detection of *ARF17* (*Solyc11g013470-80*), *ARF2A* (*Solyc03g118290*), and *ASR4* (*Solyc04g071620.2*) gene expression levels. The value of wild type buds was set to 1.0 for each gene. To normalize expression levels, Sl-Actin-51 was amplified as an internal control. Values are means and SD ($n = 4$).

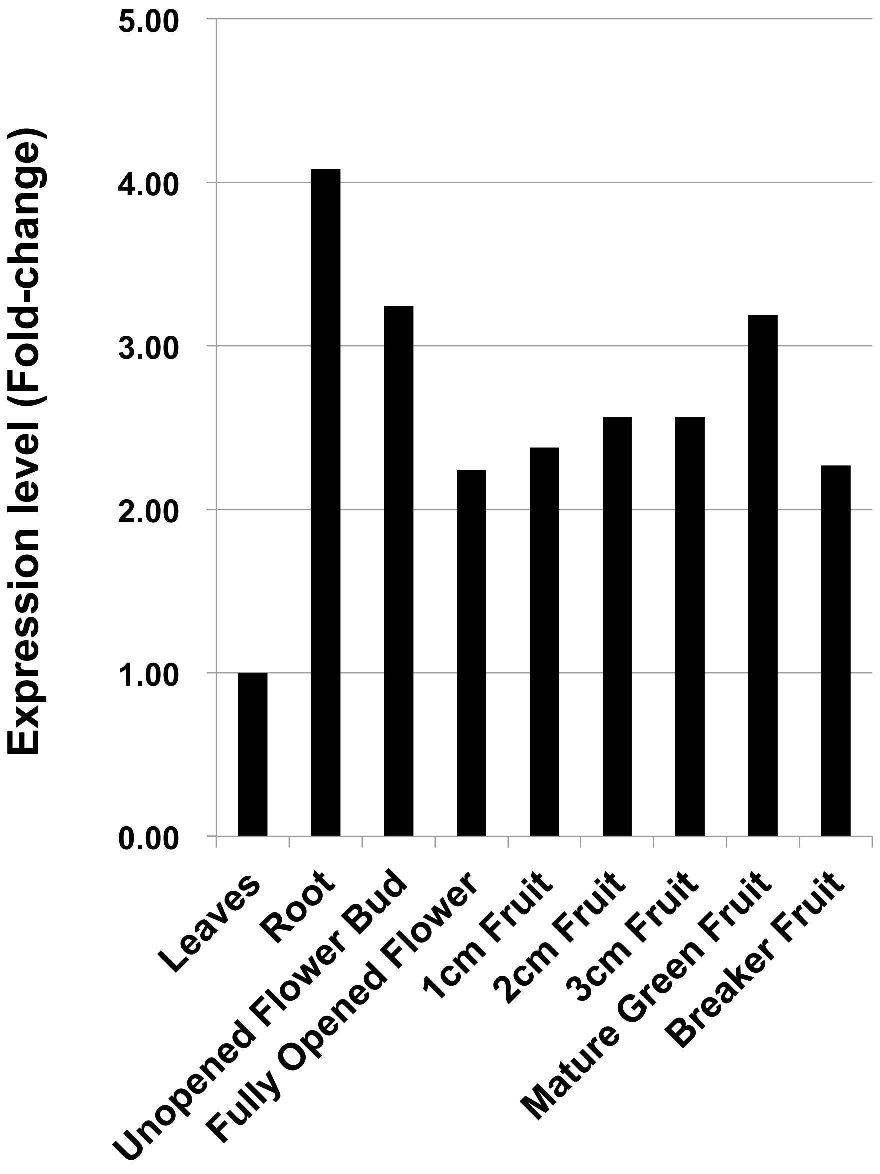

Supplementary Figure 8. Expression levels of *SlIAA9* in tomato plant tissues.

RPKM (Reads Per kb per Million reads) from RNA-seq analysis for the gene expression of *SlIAA9* (Solyc04g076850) were obtained with the aid of the Tomato eFP Browser (http://bar.utoronto.ca/efp_tomato/cgi-bin/efpWeb.cgi; Data set in the Tomato eFP Browser were from The Tomato Genome Consortium. The tomato genome sequence provides insights into fleshy fruit evolution. Nature, 485, 635-641 (2012).). The values are expressed as fold changes relative to the expression in leaves.

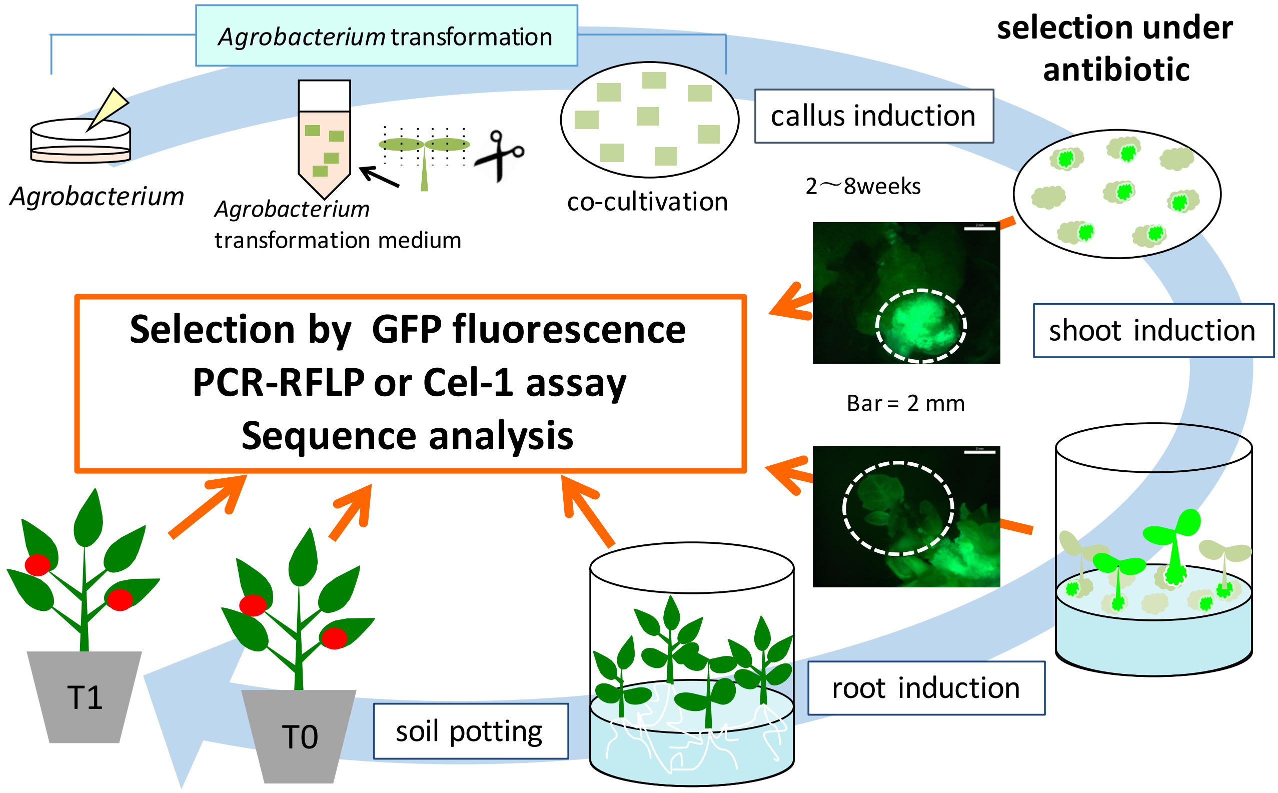

Supplementary Figure 9. Scheme illustrating production of the *SlIAA9* knockout tomato. Transgenic tomato plants with the CRISPR/Cas9 vectors introduced were generated by the *Agrobacterium*-mediated leaf disk method. Transgenic calli, shoots, and regenerated plants were selected by both antibiotic resistance and GFP fluorescence.
